# Bis[4-(dimethyl­amino)­pyridinium] dibromidodichloridodimethyl­stannate(IV)

**DOI:** 10.1107/S1600536808013561

**Published:** 2008-05-10

**Authors:** Kong Mun Lo, Seik Weng Ng

**Affiliations:** a Department of Chemistry, University of Malaya, 50603 Kuala Lumpur, Malaysia

## Abstract

The tin(IV) atom in the salt, (C_7_H_11_N_2_)_2_[SnBr_2_(CH_3_)_2_Cl_2_], lies on a center of inversion in a tetra­gonally compressed octa­hedron; the bromine atoms are disordered with the chlorine atoms, so that they appear to share the same site. The crystal structure is stabilized by N—H⋯Br hydrogen bonds.

## Related literature

For the structure of bis­(4-dimethyl­amino­pyridinium) tetra­bromidodiphenyl­stannate(IV), see: Yap *et al.* (2008 ▶).
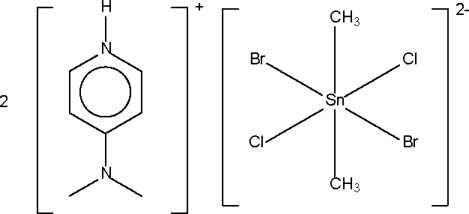


## Experimental

### 

#### Crystal data

(C_7_H_11_N_2_)_2_[SnBr_2_(CH_3_)_2_Cl_2_]
*M* *_r_* = 625.83
Triclinic, 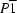

*a* = 7.3573 (3) Å
*b* = 8.7717 (3) Å
*c* = 9.6644 (4) Å
α = 97.183 (3)°
β = 107.990 (3)°
γ = 90.052 (2)°
*V* = 588.04 (4) Å^3^
*Z* = 1
Mo *K*α radiation
μ = 4.72 mm^−1^
*T* = 100 (2) K
0.25 × 0.20 × 0.15 mm

#### Data collection

Bruker SMART APEX diffractometer
Absorption correction: multi-scan (*SADABS*;Sheldrick, 1996 ▶) *T*_min_ = 0.385, *T*_max_ = 0.538 (expected range = 0.353–0.493)
4965 measured reflections
2756 independent reflections
1656 reflections with *I* > 2σ(*I*)
*R*_int_ = 0.045

#### Refinement

*R*[*F* ^2^ > 2σ(*F* ^2^)] = 0.046
*wR*(*F* ^2^) = 0.123
*S* = 1.04
2756 reflections
122 parameters
4 restraints
H-atom parameters constrained
Δρ_max_ = 0.82 e Å^−3^
Δρ_min_ = −1.14 e Å^−3^

### 

Data collection: *APEX2* (Bruker, 2007 ▶); cell refinement: *SAINT* (Bruker, 2007 ▶); data reduction: *SAINT*; program(s) used to solve structure: *SHELXS97* (Sheldrick, 2008 ▶); program(s) used to refine structure: *SHELXL97* (Sheldrick, 2008 ▶); molecular graphics: *X-SEED* (Barbour, 2001 ▶); software used to prepare material for publication: *publCIF* (Westrip, 2008 ▶).

## Supplementary Material

Crystal structure: contains datablocks global, I. DOI: 10.1107/S1600536808013561/bt2709sup1.cif

Structure factors: contains datablocks I. DOI: 10.1107/S1600536808013561/bt2709Isup2.hkl

Additional supplementary materials:  crystallographic information; 3D view; checkCIF report

## Figures and Tables

**Table 1:** Selected bond lengths (Å)

Sn1—C1	2.225 (5)
Sn1—*X*1	2.690 (1)
Sn1—*X*2	2.6926 (8)

*X* is a disordered mixture of Cl and Br.

**Table 2:** Hydrogen-bond geometry (Å, °)

*D*—H⋯*A*	*D*—H	H⋯*A*	*D*⋯*A*	*D*—H⋯*A*
N1—H1⋯*X*1	0.88	2.60	3.316 (5)	139
N1—H1⋯*X*2^i^	0.88	2.81	3.458 (6)	132

*X* is a disordered mixture of Cl and Br. Symmetry code: (i) 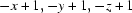.

**Fig. 1.**
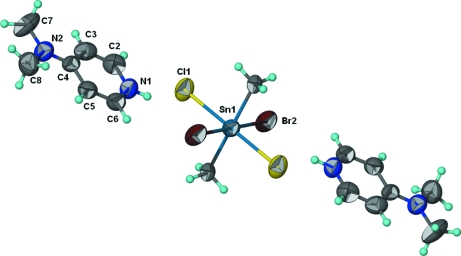
Thermal ellipsoid plot (Barbour, 2001) plot of [C7H11N]2 [SnBr2Cl2(CH3)2] at the 70% probability level. Hydrogen atoms are drawn as spheres of arbitrary radius. The disorder is not shown.

## supplementary crystallographic information

### Comment

Bis(4-methylaminopyridinium) tetrabromidodiphenylstannate is produced from the cleavage of the mixed alkyl/triarylstannate, cyclopentyltriphenyltin, by 4-dimethylaminopyridine hydrobromide perbromide (Yap *et al.*, 2008). In principle, the salt can be synthesized from the reaction of 4-dimethylaminopyridine hydrobromide perbromide and diphenyltin dibromide. The possibility is borne out by reacting the organic reagent with dimethyltin dichloride to yield the title salt (Scheme I, Fig. 1). The Sn^IV^ atom of the stannate lies on a center-of-inversion in tetragonally compressed octahedron; the two indepedent bromine atom share the sames site as the two independent chlorine atoms.

### Experimental

Dimethyltin dichoride (2.20 g, 1 mmol) and 4-dimethylaminopyridine hydrobromide perbromide (3.62 g, 1 mmol) were heated in ethanol in an attempt to synthesize the bromodichloridodimethylstannate salt. Colorless crystals separated from it after a few days.

### Refinement

Carbon-bound H-atoms were placed in calculated positions (C—H 0.95 to 0.98 Å) and were included in the refinement in the riding model approximation, with *U*(H) set to 1.2 to 1.5*U*_eq_(C). The ammonium H atom was similarly treated (N–H 0.88 Å; *U*(H) = 1.2  *U*_eq_(N)).

The chlorine atoms are disordered with respect to the bromine atoms, so that the halogen site is occupied by both a chlorine and a bromine. Constraints were applied so that at each site, the atoms had the same coordinates and the same anisotropic displacement parameters. The occupancies   refined to 0.4551 (15) for the Br1/Cl2 pair, and to  0.5449 (15)   for the Br2/Cl1 pair. The final difference Fourier map had a large peak at 1 Å from Sn1.

### Crystal data

**Table:**

(C_7_H_11_N_2_)_2_[SnBr_2_(CH_3_)_2_Cl_2_]	*Z* = 1
*M**_r_* = 625.83	*F*_000_ = 306
Triclinic, *P*1	*D*_x_ = 1.767 Mg m^−^^3^
Hall symbol: -P 1	Mo *K*α radiation λ = 0.71073 Å
*a* = 7.3573 (3) Å	Cell parameters from 1302 reflections
*b* = 8.7717 (3) Å	θ = 2.3–23.1º
*c* = 9.6644 (4) Å	µ = 4.72 mm^−^^1^
α = 97.183 (3)º	*T* = 100 (2) K
β = 107.990 (3)º	Prism, colorless
γ = 90.052 (2)º	0.25 × 0.20 × 0.15 mm
*V* = 588.04 (4) Å^3^	

### Data collection

**Table:**

Bruker SMART APEX diffractometer	2756 independent reflections
Radiation source: fine-focus sealed tube	1656 reflections with *I* > 2σ(*I*)
Monochromator: graphite	*R*_int_ = 0.045
*T* = 100(2) K	θ_max_ = 27.5º
ω scans	θ_min_ = 2.3º
Absorption correction: Multi-scan(SADABS;Sheldrick, 1996)	*h* = −9→9
*T*_min_ = 0.385, *T*_max_ = 0.538	*k* = −11→11
4965 measured reflections	*l* = −10→12

### Refinement

**Table:**

Refinement on *F*^2^	Secondary atom site location: difference Fourier map
Least-squares matrix: full	Hydrogen site location: inferred from neighbouring sites
*R*[*F*^2^ > 2σ(*F*^2^)] = 0.046	H-atom parameters constrained
*wR*(*F*^2^) = 0.123	*w* = 1/[σ^2^(*F*_o_^2^) + (0.051*P*)^2^] where *P* = (*F*_o_^2^ + 2*F*_c_^2^)/3
*S* = 1.04	(Δ/σ)_max_ = 0.001
2756 reflections	Δρ_max_ = 0.82 e Å^−^^3^
122 parameters	Δρ_min_ = −1.14 e Å^−^^3^
4 restraints	Extinction correction: none
Primary atom site location: structure-invariant direct methods	

### Fractional atomic coordinates and isotropic or equivalent isotropic displacement parameters (Å^2^)

**Table:**

	*x*	*y*	*z*	*U*_iso_*/*U*_eq_	Occ. (<1)
Sn1	0.5000	0.5000	0.5000	0.0414 (2)	
Br1	0.50474 (16)	0.50403 (11)	0.77987 (11)	0.0610 (3)	0.4551 (15)
Br2	0.36593 (14)	0.78543 (10)	0.49452 (10)	0.0588 (3)	0.5449 (15)
Cl1	0.50474 (16)	0.50403 (11)	0.77987 (11)	0.0610 (3)	0.5449 (15)
Cl2	0.36593 (14)	0.78543 (10)	0.49452 (10)	0.0588 (3)	0.4551 (15)
N1	0.6576 (7)	0.1602 (6)	0.8588 (6)	0.0552 (14)	
H1	0.6205	0.2248	0.7938	0.066*	
N2	0.8414 (7)	−0.1391 (6)	1.1635 (6)	0.0557 (14)	
C1	0.2014 (7)	0.4016 (6)	0.4157 (6)	0.0344 (12)	
H1A	0.2023	0.2904	0.4192	0.052*	
H1B	0.1432	0.4214	0.3142	0.052*	
H1C	0.1271	0.4495	0.4767	0.052*	
C2	0.6816 (9)	0.2092 (8)	1.0006 (8)	0.0581 (18)	
H2	0.6571	0.3128	1.0290	0.070*	
C3	0.7413 (9)	0.1116 (7)	1.1061 (7)	0.0545 (16)	
H3	0.7570	0.1480	1.2060	0.065*	
C4	0.7793 (8)	−0.0433 (7)	1.0650 (7)	0.0443 (15)	
C5	0.7486 (8)	−0.0875 (7)	0.9140 (7)	0.0488 (15)	
H5	0.7698	−0.1904	0.8805	0.059*	
C6	0.6890 (9)	0.0144 (8)	0.8144 (7)	0.0536 (16)	
H6	0.6698	−0.0183	0.7132	0.064*	
C7	0.8687 (11)	−0.0920 (10)	1.3200 (8)	0.084 (3)	
H7A	0.9886	−0.0305	1.3639	0.127*	
H7B	0.8740	−0.1836	1.3695	0.127*	
H7C	0.7616	−0.0305	1.3310	0.127*	
C8	0.8809 (10)	−0.2972 (8)	1.1236 (9)	0.071 (2)	
H8A	0.7602	−0.3560	1.0736	0.106*	
H8B	0.9498	−0.3417	1.2124	0.106*	
H8C	0.9594	−0.3008	1.0579	0.106*	

### Atomic displacement parameters (Å^2^)

**Table:**

	*U*^11^	*U*^22^	*U*^33^	*U*^12^	*U*^13^	*U*^23^
Sn1	0.0437 (4)	0.0408 (3)	0.0388 (4)	0.0028 (3)	0.0122 (3)	0.0040 (3)
Br1	0.0938 (9)	0.0501 (6)	0.0460 (6)	0.0082 (5)	0.0319 (6)	0.0062 (5)
Br2	0.0809 (7)	0.0458 (5)	0.0461 (6)	0.0187 (5)	0.0144 (5)	0.0062 (4)
Cl1	0.0938 (9)	0.0501 (6)	0.0460 (6)	0.0082 (5)	0.0319 (6)	0.0062 (5)
Cl2	0.0809 (7)	0.0458 (5)	0.0461 (6)	0.0187 (5)	0.0144 (5)	0.0062 (4)
N1	0.050 (3)	0.064 (4)	0.052 (4)	0.001 (3)	0.012 (3)	0.020 (3)
N2	0.048 (3)	0.070 (4)	0.050 (3)	0.004 (3)	0.012 (3)	0.017 (3)
C1	0.032 (3)	0.039 (3)	0.040 (3)	0.009 (2)	0.019 (3)	0.012 (3)
C2	0.050 (4)	0.058 (4)	0.064 (5)	0.007 (3)	0.016 (4)	0.004 (4)
C3	0.048 (4)	0.065 (4)	0.048 (4)	0.001 (3)	0.016 (3)	−0.006 (3)
C4	0.031 (3)	0.063 (4)	0.037 (3)	−0.005 (3)	0.008 (3)	0.009 (3)
C5	0.047 (4)	0.050 (3)	0.047 (4)	−0.003 (3)	0.013 (3)	0.002 (3)
C6	0.054 (4)	0.064 (4)	0.042 (4)	−0.013 (3)	0.016 (3)	0.001 (3)
C7	0.075 (5)	0.136 (7)	0.043 (4)	0.025 (5)	0.013 (4)	0.028 (5)
C8	0.071 (5)	0.063 (5)	0.079 (6)	0.002 (4)	0.016 (4)	0.026 (4)

### Geometric parameters (Å, °)

**Table:**

Sn1—C1^i^	2.225 (5)	C1—H1C	0.9800
Sn1—C1	2.225 (5)	C2—C3	1.381 (8)
Sn1—Br1	2.690 (1)	C2—H2	0.9500
Sn1—Cl1^i^	2.690 (1)	C3—C4	1.420 (9)
Sn1—Br1^i^	2.690 (1)	C3—H3	0.9500
Sn1—Br2^i^	2.6926 (8)	C4—C5	1.409 (9)
Sn1—Br2	2.6926 (8)	C5—C6	1.369 (8)
Sn1—Cl2^i^	2.6926 (8)	C5—H5	0.9500
N1—C2	1.341 (9)	C6—H6	0.9500
N1—C6	1.341 (9)	C7—H7A	0.9800
N1—H1	0.8800	C7—H7B	0.9800
N2—C4	1.324 (7)	C7—H7C	0.9800
N2—C8	1.446 (9)	C8—H8A	0.9800
N2—C7	1.467 (9)	C8—H8B	0.9800
C1—H1A	0.9800	C8—H8C	0.9800
C1—H1B	0.9800		
			
C1^i^—Sn1—C1	180.0	Sn1—C1—H1A	109.5
C1^i^—Sn1—Br1	88.38 (14)	Sn1—C1—H1B	109.5
C1—Sn1—Br1	91.62 (14)	H1A—C1—H1B	109.5
C1^i^—Sn1—Cl1^i^	91.62 (14)	Sn1—C1—H1C	109.5
C1—Sn1—Cl1^i^	88.38 (14)	H1A—C1—H1C	109.5
Br1—Sn1—Cl1^i^	180.0	H1B—C1—H1C	109.5
C1^i^—Sn1—Br1^i^	91.62 (14)	N1—C2—C3	121.1 (6)
C1—Sn1—Br1^i^	88.38 (14)	N1—C2—H2	119.5
Br1—Sn1—Br1^i^	180.0	C3—C2—H2	119.5
Cl1^i^—Sn1—Br1^i^	0.0	C2—C3—C4	120.0 (6)
C1^i^—Sn1—Br2^i^	89.83 (13)	C2—C3—H3	120.0
C1—Sn1—Br2^i^	90.17 (13)	C4—C3—H3	120.0
Br1—Sn1—Br2^i^	89.12 (3)	N2—C4—C5	122.5 (6)
Cl1^i^—Sn1—Br2^i^	90.88 (3)	N2—C4—C3	121.6 (6)
Br1^i^—Sn1—Br2^i^	90.88 (3)	C5—C4—C3	115.9 (6)
C1^i^—Sn1—Br2	90.17 (13)	C6—C5—C4	121.5 (6)
C1—Sn1—Br2	89.83 (13)	C6—C5—H5	119.2
Br1—Sn1—Br2	90.88 (3)	C4—C5—H5	119.2
Cl1^i^—Sn1—Br2	89.12 (3)	N1—C6—C5	120.4 (6)
Br1^i^—Sn1—Br2	89.12 (3)	N1—C6—H6	119.8
Br2^i^—Sn1—Br2	180.0	C5—C6—H6	119.8
C1^i^—Sn1—Cl2^i^	89.83 (13)	N2—C7—H7A	109.5
C1—Sn1—Cl2^i^	90.17 (13)	N2—C7—H7B	109.5
Br1—Sn1—Cl2^i^	89.12 (3)	H7A—C7—H7B	109.5
Cl1^i^—Sn1—Cl2^i^	90.88 (3)	N2—C7—H7C	109.5
Br1^i^—Sn1—Cl2^i^	90.88 (3)	H7A—C7—H7C	109.5
Br2^i^—Sn1—Cl2^i^	0.000 (13)	H7B—C7—H7C	109.5
Br2—Sn1—Cl2^i^	180.0	N2—C8—H8A	109.5
C2—N1—C6	121.1 (6)	N2—C8—H8B	109.5
C2—N1—H1	119.4	H8A—C8—H8B	109.5
C6—N1—H1	119.4	N2—C8—H8C	109.5
C4—N2—C8	122.3 (6)	H8A—C8—H8C	109.5
C4—N2—C7	121.8 (6)	H8B—C8—H8C	109.5
C8—N2—C7	115.9 (6)		
			
C6—N1—C2—C3	0.6 (9)	C2—C3—C4—N2	178.3 (5)
N1—C2—C3—C4	0.3 (10)	C2—C3—C4—C5	−1.1 (8)
C8—N2—C4—C5	−0.6 (9)	N2—C4—C5—C6	−178.3 (6)
C7—N2—C4—C5	−178.7 (6)	C3—C4—C5—C6	1.1 (9)
C8—N2—C4—C3	−180.0 (5)	C2—N1—C6—C5	−0.7 (9)
C7—N2—C4—C3	1.9 (9)	C4—C5—C6—N1	−0.2 (9)

Symmetry codes: (i) −*x*+1, −*y*+1, −*z*+1.

### Hydrogen-bond geometry (Å, °)

**Table:**

*D*—H···*A*	*D*—H	H···*A*	*D*···*A*	*D*—H···*A*
N1—H1···Br1	0.88	2.60	3.316 (5)	139
N1—H1···Br2^i^	0.88	2.81	3.458 (6)	132

Symmetry codes: (i) −*x*+1, −*y*+1, −*z*+1.
